# Primary Central Nervous System Lymphoma Presenting as a Solitary Fourth Ventricular Mass: A Case Report

**DOI:** 10.7759/cureus.66486

**Published:** 2024-08-09

**Authors:** Dima Abu Laban, Bayan Maraqa, Alaa Abufara, Abdullah Nofal, Akram Al-Ibraheem

**Affiliations:** 1 Diagnostic Radiology, King Hussein Cancer Center (KHCC), Amman, JOR; 2 Pathology, King Hussein Cancer Center (KHCC), Amman, JOR; 3 Medical Oncology, King Hussein Cancer Center (KHCC), Amman, JOR; 4 Nuclear Medicine, King Hussein Cancer Center (KHCC), Amman, JOR

**Keywords:** rare brain tumor, mri, fourth ventricle, pcnsl, primary central nervous system lymphoma (pcnsl), cns lymphoma, lymphoma, cns

## Abstract

The occurrence of primary fourth ventricular lymphoma is an exceptionally uncommon phenomenon. Here, we present a case of lymphoma in the fourth ventricle in a 30-year-old male who presented with progressive headache and vertigo over the last one month of his presentation. Preoperative MRI revealed a space-occupying lesion of the fourth ventricle. Pathological analysis following complete resection confirmed the lesion as primary central nervous system lymphoma. The patient underwent chemotherapy following the MTR (methotrexate, temozolomide, and rituximab) protocol with four months of uneventful follow-up, indicating no disease recurrence. Therefore, clinicians are advised to consider the potential presence of lymphoma as part of the differential diagnosis for space-occupying lesions, especially when there is a combination of clinical deterioration and rapid imaging progression.

## Introduction

Primary central nervous system lymphoma (PCNSL) is defined as an extranodal lymphomatous disease that is confined to the central nervous system (CNS). It is a rare subtype of lymphoma, comprising only a small percentage (1%-5%) of all brain tumors [1]. The majority of PCNSL cases fall under the category of non-Hodgkin's lymphoma (NHL), representing just 1% of all NHL cases [2]. In contrast, secondary CNS lymphoma, which involves the spread of lymphoma from other parts of the body to the CNS, is more common than primary CNS lymphoma [3].

In contrast to secondary lymphoma, which may affect the meninges, PCNSL typically presents as supratentorial lesions within the brain parenchyma [4]. The localization of infratentorial lesions is rarely observed, with the majority of cases being found within the cerebellum. In contrast, spinal and brainstem localization is an exceedingly uncommon site for PCNSL [4]. Individuals who are immunocompromised, such as those infected with human immunodeficiency virus (HIV), have an increased risk of developing PCNSL [5]. However, the incidence of PCNSL in HIV-positive individuals has decreased with the widespread use of highly active antiretroviral therapy [6]. Conversely, the prevalence of PCNSL is on the rise in immunocompetent individuals [7].

In the context of PCNSL, the occurrence of intraventricular lesions is uncommon, and there have been fewer than 30 documented cases specifically involving lesions in the fourth ventricle [8,9]. A thorough comprehension of the clinical and radiological manifestations of PCNSL is essential for accurate diagnosis due to the presence of numerous differential diagnoses. PCNSL typically presents as a a solitary lesion, often in contact with ventricular or meningeal surfaces, with hyper- or isoattenuation on CT [4]. On MRI, PCNSL lesions are hypo- or isointense on T1-weighted images and iso- to hyperintense (often hypointense to gray matter) on T2-weighted images, showing moderate-to-marked, usually homogeneous enhancement. Hemorrhage or calcification is rare. In contrast, secondary CNS lymphoma often presents with multiple, more diffusely infiltrative lesions, frequently involving the leptomeninges, cranial nerves, and spinal cord, with variable CT and MRI characteristics, including more heterogeneous enhancement and a higher likelihood of hemorrhage or calcification, especially in treated cases [4]. This case report highlights a unique occurrence of PCNSL in the fourth ventricle, which displayed rapid morphological changes and clinical deterioration.

## Case presentation

**Figure 1:**
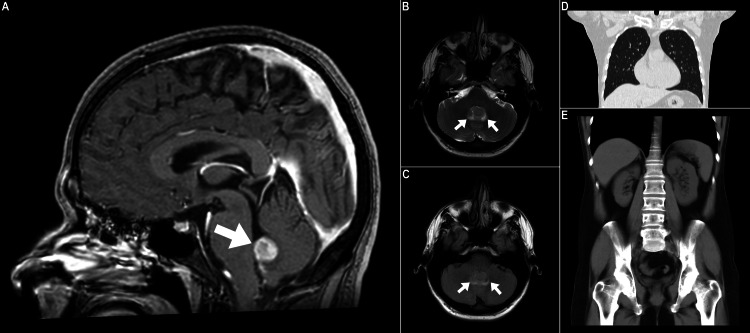
Baseline brain MRI. (A) Initial sagittal T1-weighted image performed post-gadolinium contrast medium administration, (B) T2-weighted axial view, and (C) fluid attenuation inversion recovery (FLAIR) axial brain MRI sequences. The study revealed a fourth ventricular mass measuring 1.5 cm in maximum dimensions (arrows), and demonstrating contrast enhancement with intermediate signal on T2-weighted and FLAIR sequences. Notably, there is very subtle edema surrounding the fourth ventricle. (D, E) Coronal chest, abdomen, and pelvic CT views were unremarkable for any concerning extracranial neoplastic lesions.

A 30-year-old previously healthy male with no prior history of surgical procedures and no family history of malignancies presented with symptoms of morning headaches, dizziness, and nausea over two weeks. Initially, it was thought to be due to visual impairment. Ophthalmologic examination revealed intact visual acuity and excluded papilledema. Brain MRI identified a single intra-ventricular mass in the fourth ventricle, prompting consideration of various differential diagnoses including sub-ependymoma, ependymoma, choroid plexus papilloma, hemangioblastoma, meningioma, and glioma (Figure 1A-1C). It is worth noting that a chest, abdomen, and pelvic CT was offered during the same timeframe and was unremarkable for any concerning extracranial neoplastic lesions (Figure 1D, 1E).

**Figure 2:**
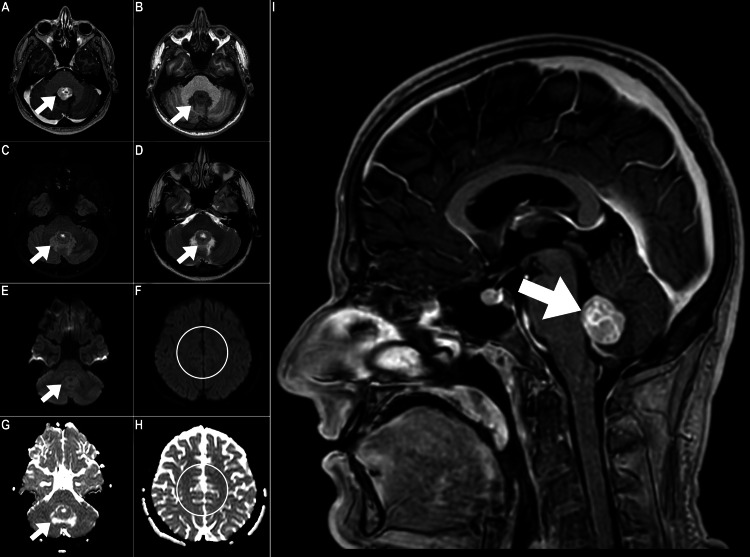
Follow-up brain MRI. (A) Follow-up axial T1-weighted image post gadolinium contrast medium administration, (B) axial T1-weighted pre-gadolinium contrast medium administration, (C) fluid attenuation inversion recovery (FLAIR) axial sequence, (D) T2-weighted axial brain MRI sequence, (E, F) diffusion-weighted images, (G, H) apparent diffusion coefficient maps, and (I) sagittal T1-weighted image post-gadolinium contrast medium administration was performed three weeks later. The mass within the fourth ventricle shows an intermediate signal on T1- and T2-weighted sequences with avid contrast enhancement and a central small cystic component. The mass shows marked enlargement over short intervals (measuring about 3 cm vs 1.5 cm previously) with moderate edema surrounding the fourth ventricle appreciated on T2-weighted and FLAIR sequences.  No proximal ventricle dilatation was seen. The diffusion-weighted images and apparent diffusion coefficient map at the lesion and centrum semiovale showed no diffusion restriction within the mass.

Three weeks afterward, the patient sought oncologic consultation in our cancer center and reported worsened headaches, frequent vomiting, and dizziness. A comprehensive evaluation, including clinical, neurological, and biochemical examinations, revealed no significant findings. A follow-up brain MRI was performed due to the patient's complaints. The MRI results indicated an enlargement of the CNS mass, accompanied by an increase in the surrounding edema (Figure 2). Notably, no diffusion restriction was observed in diffusion-weighted images (Figure 2E-2H).

**Figure 3:**
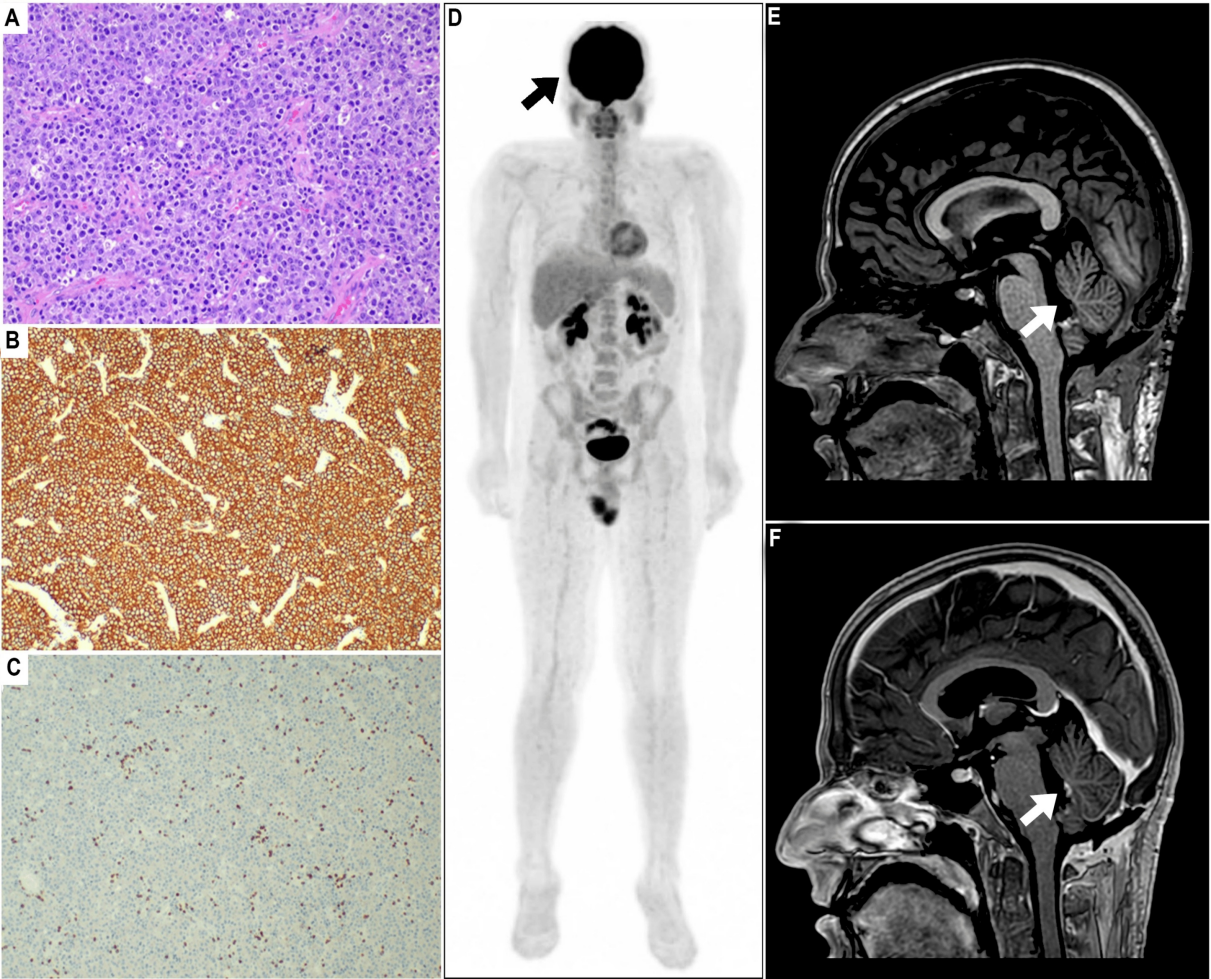
Evaluation post-surgery. (A) Histopathologic examination revealed a diffuse infiltrate of atypical lymphoid cells with vesicular chromatin and prominent nucleoli. (B, C) Immunohistochemistry analysis demonstrates positive expression for (B) CD20 and negative expression for (C) CD3. A KI-67 index of 90% was reported. (D) Maximum intensity projection image of 18F-fluorodeoxyglucose (FDG) PET/CT was unremarkable for systemic lymphoma. (E, F) Postoperative sagittal T1-weighted images conducted (E) before and (F) after gadolinium contrast medium administration showed gross total resection of the mass.

Importantly, no additional intracranial mass or proximal hydrocephalus was observed. A midline suboccipital craniotomy was performed. Intraoperatively, a soft pale non-hemorrhagic mass was identified adherent to the roof of the fourth ventricle. The tumor was successfully excised, leading to a histopathological diagnosis of stage IE intraventricular lymphoma, specifically diffuse large B cell lymphoma (Figure 3A-3C).

Further imaging with 18F-fluorodeoxyglucose (FDG) PET/CT excluded systemic lymphoma (Figure 3D). A multidisciplinary team discussion was initiated following the surgery to determine the best next line of therapy. The team opted for chemotherapy-only treatment as per institutional guidelines adhering to MTR (methotrexate, temozolomide, and rituximab) protocol. A total of eight chemotherapy cycles was offered with four months of uneventful follow-up, indicating no disease recurrence (Figure 3E, 3F).

## Discussion

PCNSL is an uncommon condition, accounting for a small proportion of brain and spinal cord tumors [10]. It refers to the presence of lymphoma in the CNS without any signs of the disease affecting other parts of the body at the time of diagnosis [10]. Individuals who have a weakened immune system due to HIV infection are recognized to have a higher likelihood of experiencing health complications [5]. Nevertheless, the prevalence of the disease has diminished in the aforementioned population following the implementation of highly active antiretroviral therapy [6]. Conversely, there is a growing occurrence of the disease in patients with a fully functioning immune system [7]. PCNSL lesions primarily develop in the brain parenchyma, and there have been fewer than 30 documented cases of a single CNS lymphoma occurring in the fourth ventricle [9]. 

The initial MRI images of our patient indicated the presence of an enhancing sub-ependymoma or intraventricular meningioma, along with ependymoma. The subsequent examination revealed an increasing mass, raising concerns for a malignant condition. Kojima et al. documented the 18th instance of diffuse large B cell lymphoma, which is the most common form of the disease and typically affects individuals in their 50s and 60s [11]. This finding was supported by a review of existing literature [11]. Two additional cases, as reported by Holanda et al. and Zhao et al., were observed in a 45-year-old male and a 48-year-old male, respectively [6,12]. There is a higher proportion of males affected, with 16 cases occurring in males compared to only five cases reported in females. Two instances of Burkitt lymphoma were reported in male patients aged 18 years and 32 years, respectively [13,14]. Notably, choroid plexus tumors are a potential differential diagnosis to consider; however, they are primarily seen in pediatric populations, making their occurrence in adulthood highly improbable [15].

For adults, the differential diagnosis includes ependymoma, choroid plexus papilloma, intraventricular hemangioblastoma, meningioma, or medulloblastoma [6]. These lesions are not anticipated to exhibit rapid growth within a short timeframe [16]. The progressive enlargement of the lesion over three weeks was concerning, leading to the decision to perform surgery. Accurately assessing treatment recommendations is challenging due to the limited number of patients. Nevertheless, the majority of patients underwent total excision, followed by chemoradiotherapy [17].

PCNSL exhibits a significant initial response to chemoradiation treatment. However, the overall survival rate is still unsatisfactory [18]. As per the findings of the German PCNSL study group 1, patients who had a subtotal or gross total resection had a longer period without disease progression compared to patients who only had a biopsy [19].

## Conclusions

Encountering primary central nervous system lymphoma (PCNSL) within the fourth ventricle is highly uncommon. In cases where a lesion exhibits rapid growth within a brief timeframe, clinicians are advised to consider the potential presence of PCNSL as part of the differential diagnosis. Histopathological analysis plays a vital role in confirming the diagnosis. Furthermore, supplementary imaging assessments such as 18F-fluorodeoxyglucose (FDG) PET/CT can be utilized to exclude the existence of any systemic lymphomatous disease. Utilizing this approach is vital for ensuring accurate disease staging and adherence to the proper treatment procedures.
